# Supplementary material for: Efficacy of a Chitin-Based Water-Soluble Derivative in Inducing Purpureocillium lilacinum against Nematode Disease (Meloidogyne incognita)
Source: Int J Mol Sci. 2021 Jun 26;22(13):6870. doi: 10.3390/ijms22136870 (PMC8268436; doi:10.3390/ijms22136870)
Supplement: Supplementary file 1 [file ijms-22-06870-s001.zip › ijms-1235047-SI.pdf]

# Appendix A. Supplementary data

## Efficacy of chitin-based water-soluble derivative as an environmental nematicidal candidate in inducing *Paecilomyces lilacinus* against nematode disease (*Meloidogyne incognita*)

***Table S 1*** *The physical and chemical properties of 6-oxychitin*

| Sample | Water solubility (mg/ml) | Mw (Da) | OD (%) | Carboxylate content(mmol/g) |
|---|---|---|---|---|
| 6-Oxychitin | 26.57 | 2100 | 16.28 | 0.974 |

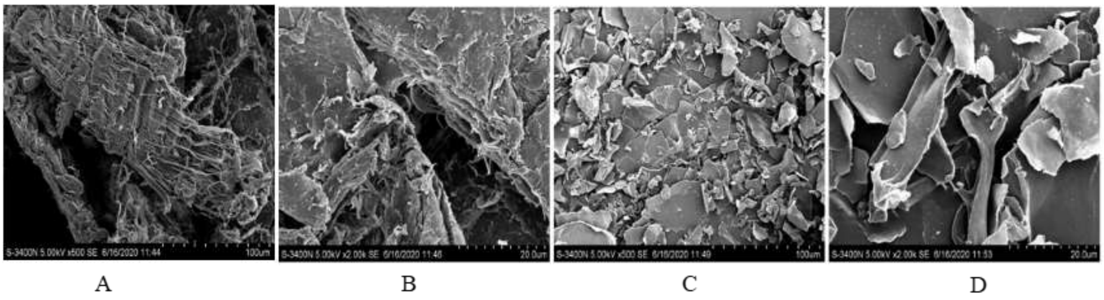

***Figure S 1*** *The morphology of chitin (A、B) and oxidized chitin (C、D) under Scanning Electron Microscope. A and C are at 500 times magnification; B and D are at 2000 times magnification.*
